# Supplementary material for: Scalable trapping of single nanosized extracellular vesicles using plasmonics
Source: Nat Commun. 2023 Aug 9;14:4801. doi: 10.1038/s41467-023-40549-7 (PMC10412615; doi:10.1038/s41467-023-40549-7)
Supplement: Supplementary file 1 — Supplementary Information [file 41467_2023_40549_MOESM1_ESM.pdf]

Scalable trapping of single nanosized extracellular vesicles using plasmonics

Chuchuan Hong,[1,2], Justus C. Ndukaife[1,2,3*]

[1]Department of Electrical and Computer Engineering, Vanderbilt University, Nashville, TN, USA
[2]Vanderbilt Institute of Nanoscale Science and Engineering, Vanderbilt University, Nashville, TN, USA
[3]Department of Mechanical Engineering, Vanderbilt University, Nashville, TN, USA
*justus.ndukaife@vanderbilt.edu

Supplementary Discussion I. Generation of opposing AC Electro-osmotic flow in GET

We solved the Poisson equation below to calculate the electric field distribution near the plasmonic nanohole array:

$$\nabla \cdot \vec{\mathbf{E}} = \frac{\rho}{\varepsilon}, \text{ with } \vec{\mathbf{E}} = -\nabla V. \quad (1)$$

ρ is the total volume charge density, and ε is the permittivity of the medium. $\vec{\mathbf{E}}$ is the electric field and V is the electric potential energy.

The calculated electric field distribution is fed into the laminar flow simulation model for a.c. electro-osmotic flow (ACEO) simulation. The opposing ACEO flows enable the generation of the electrohydrodynamic potential for in-plane confinement of the EVs.

Firstly, the ACEO flow is obtained using the laminar flow module and prescribing a slip boundary condition given by the Smoluchowski slip velocity around the nanohole array.

The Smoluchowski slip velocity $\vec{\mathbf{u}}$ is given by: $\vec{\mathbf{u}} = \mu_{eo}\overrightarrow{\mathbf{E}_t}$, where $\mu_{eo} = -\frac{\varepsilon_r \varepsilon_0 \zeta}{\eta}$ is the electroosmotic mobility, ε_r is the relative permittivity, ε_0 is the permittivity of free-space, ζ is the zeta potential of the electrical double layer at the nanohole array and fluid interface, and η is the dynamic viscosity of the liquid. $\overrightarrow{\mathbf{E}_t} = \vec{\mathbf{E}} - (\vec{\mathbf{E}} \cdot \vec{\mathbf{n}})\vec{\mathbf{n}}$ is the tangential component of the electric field, where $\vec{\mathbf{E}}$ is calculated by solving equation 1.

The zeta potential is increasing as a.c. frequency reduces[1]. The Navier-Stokes (NS) equation is solved to find the velocity distribution and velocity vectors due to the ACEO flow. The NS equation is given by:

$$\rho_0[\vec{\mathbf{u}}(\mathbf{r}) \cdot \nabla]\vec{\mathbf{u}}(\mathbf{r}) + \nabla p(\mathbf{r}) - \eta \nabla^2 \vec{\mathbf{u}}(\mathbf{r}) = \mathbf{F} \text{ with continuity equation given by: } \rho_0\left(\nabla \cdot \vec{\mathbf{u}}(\mathbf{r})\right) = 0. \quad (2)$$

ρ_0 presents the density of liquid. $\mathbf{F}$ here is considered zero since no body force is applied. On the gold surface, a slip boundary condition is applied given by the Smoluchowski slip velocity to solve equation 2 and calculate the ACEO flow velocity. The solution was achieved by using COMSOL Multiphysics software and shows that the ACEO flows act to focuse particles at the center of the void regions.

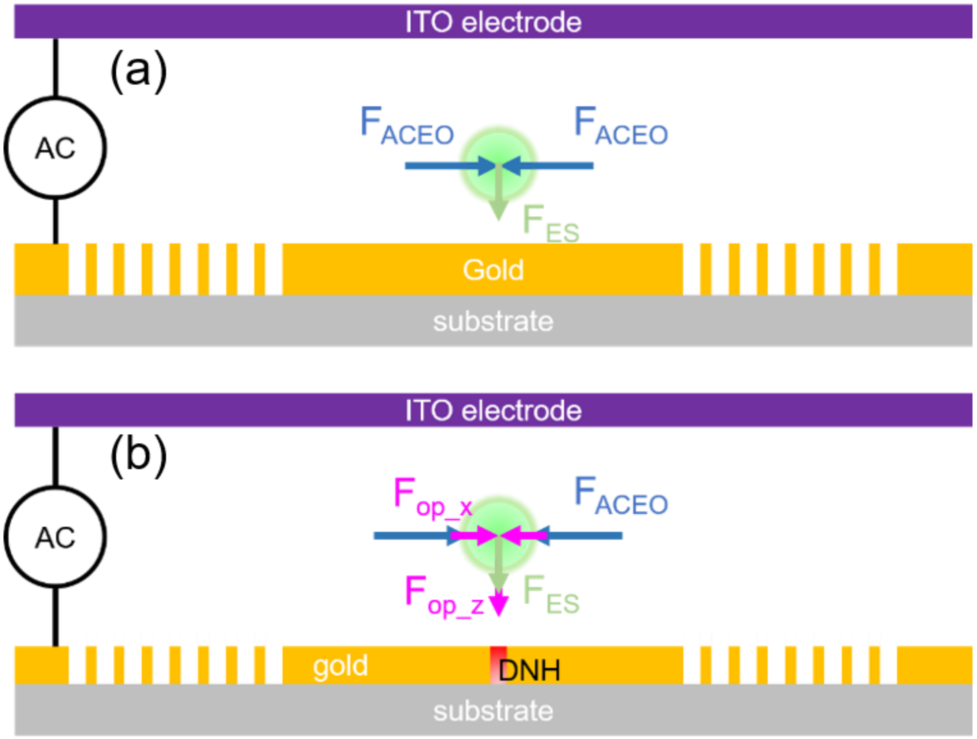

Supplementary Figure 1: force illustrations. (a) the forces acting on a trapped particle when the a.c. field is applied. (b) the forces acting when both the a.c. electric field and laser are applied. F_{ACEO} is the drag force from in-place a.c. electro-osmotic flow, F_{ES} is particle-surface interaction force, F_{op_x}, F_{op_z} is the optical gradient force along the radial and vertical directions, respectively.

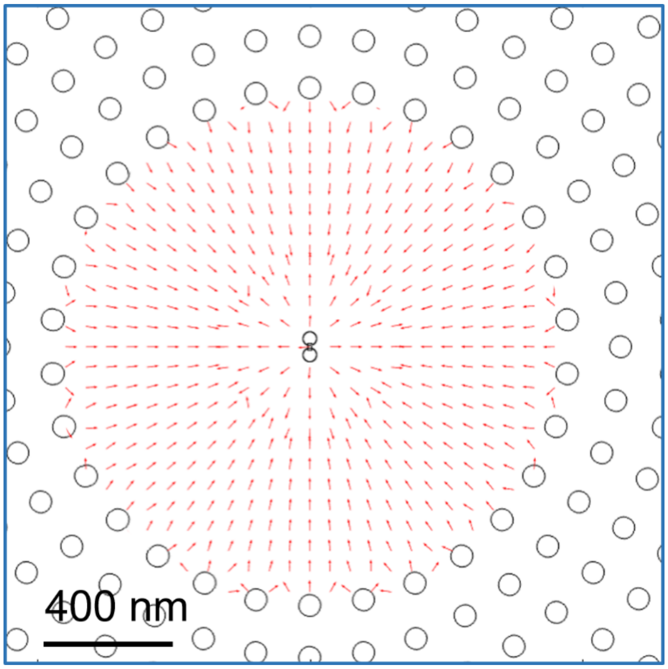

Supplementary Figure 2: in plane ACEO flow profile 10 nm above gold film with DNH, showing the DNH has its own local ACEO flow to push outwards. Thus, the trapping stability is maximized when only laser was on as shown in Fig. 4f.

Supplementary Discussion II. Simulation of dynamic relocation with electrothermoplasmonic flow

For the gold film on glass substrate, the power dissipation density in the gold film is modeled using equation 2 shown below in COMSOL Multiphysics while considering the Gaussian distribution of the incident laser beam and exponential decay of light in the gold film.

$$Q(\mathrm{r}) = P_0 A \frac{\alpha_{Au}}{\pi r^2} \mathrm{e}^{-(\frac{x^2+y^2}{2r^2})} e^{-\alpha_{Au} z} \tag{3}$$

,where P_0 is incident laser power, A is the absorption of the nanohole array. r is the beam radius. The attenuation coefficient in the nanohole array at the illumination wavelength is given by $\alpha_{Au} = \frac{4\pi\kappa_{Au}}{\lambda} = 8.91 \times 10^7\ m^{-1}$, where κ_{Au} is the imaginary part of the refractive index of gold. The lattice constant of the nanohole array is 350 nm, while the thickness of the gold film is 120 nm.

For the double nanohole on sapphire, we numerically calculate the absorption cross section of the gold region surrounding the double nanohole using Finite-Difference Time-Domain method (FDTD) and multiply it by the intensity we used in experiment to obtain the total power absorbed by double nanohole region.

The temperature distribution under laser illumination is obtained by solving the heat conduction equation given by:

$$-\mathrm{k}\nabla^2 T + \rho_0 C_p \vec{\mathbf{u}} \cdot \nabla T = Q, \tag{4}$$

k is the thermal conductivity of the materials (glass, sapphire, gold, or water). T is the temperature in Kelvin, and ∇T is the temperature gradient. The second term is the convection term, which depends on $\mathbf{u}$, the velocity of the fluid. For the chamber height in our case, 120 µm, the convection term can be neglected[2], due to the low thermal Peclet number, ρ_0 is the density of the fluid and C_p is the specific heat capacity at constant pressure. Q is the heat source density. For the case when a region of the nanohole array is illuminated, we obtain Q by solving equation 3. On the other hand when the laser is illuminated on the double nanohole aperture for plasmonic trapping, Q is obtained by multiplying the absorption cross section of the gold film region surrounding the double nanohole aperture by the laser intensity and normalizing by the volume. The outer temperature on the outer surface of the boundaries was set to $T = 293.15\ K$.

Secondly, the ETP flow arises from the localized heating of the fluid to establish a gradient in the permittivity and electrical conductivity in conjunction with an applied a.c. electric field. The volumetric body force induced in the fluid known as the electrothermal force density, $\mathbf{f}_{\mathrm{ET}}$ is given by[2]:

$$\mathbf{f}_{\mathrm{ET}} = \rho_e \vec{\mathbf{E}} - \frac{1}{2} \left|\vec{\mathbf{E}}\right|^2 \nabla \varepsilon_m. \tag{5}$$

After perturbative expansion in the limit of small temperature gradient, the force density is transformed into[3]:

$$\mathbf{f}_{\mathrm{ET}} = \frac{1}{2} \varepsilon \{ \frac{(\gamma-\beta)(\nabla T.\vec{\mathrm{E}})\vec{\mathrm{E}}}{1+(\omega\tau)^2} - \frac{1}{2} \gamma \left|\vec{\mathrm{E}}\right|^2 \nabla T \}, \tag{6}$$

where $\gamma = {}^{1}/_{\varepsilon} \frac{\mathrm{d}\varepsilon}{\mathrm{d}T} = -0.004\ \mathrm{K}^{-1}$ and $\beta = {}^{1}/_{\sigma} \frac{\mathrm{d}\sigma}{\mathrm{d}T} = 0.02\ \mathrm{K}^{-1}$. ε and σ stands for the permittivity and conductivity of the fluid, respectively. ω is the a.c. field frequency, while ∇T is the temperature gradient calculated from solving the heat equation stated in equation 4.

Subsequently, we solved the NS equation using the laminar flow module in COMSOL Multiphysics to determine the velocity. Distinct from the ACEO flow calculation, here f_{ET} is considered the body force per unit volume in the NS equation.

The aforementioned equations are solved to obtain the in-plane radial velocity vectors resulting from the induced electrothermal flow depicted in Supplementary Fig. 3 with the GET traps sitting on a sapphire substrate. This electrothermal flow acts to transport the particles from an adjacent GET trap, thus releasing the particle. Once the laser is turned OFF, the electrothermal flow disappears, and the in-plane a.c. electro-osmotic flow acts to deliver the particle to the center of the nearest GET trap. By so doing, dynamic relocation of a particle from one GET trap to the next is readily achieved.

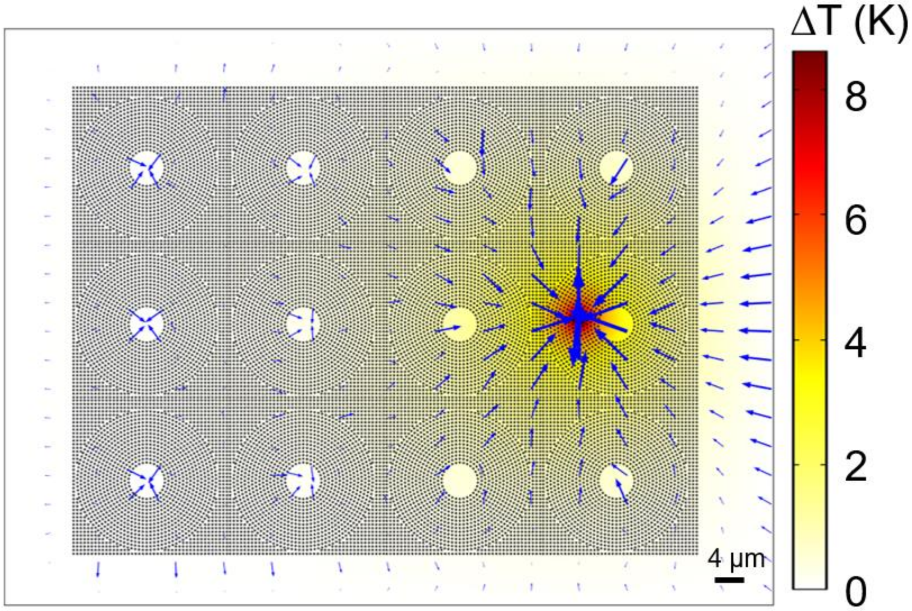

Supplementary Figure 3: thermal simulation showing the 25 mW laser focused on the gold nanohole pattern on sapphire substrate. The temperate rise is as high as 8.44 K, which is sufficient for ETP to be generated. It is evident that the ETP flow can perturb a nearby GET trap to release a particle from the GET trap and transport it. Upon turning OFF the laser, the particle can be relocated to another nearby GET trap by the ACEO flow. Blue arrows show the in-plane velocity of the supermimposed ETP and ACEO flow. The flow vectors closest to the focus of the 25 mW laser is due to the ETP flow. GET traps located farther away from the 25 mW laser spot still experience ACEO flow that can focus particles to the center.

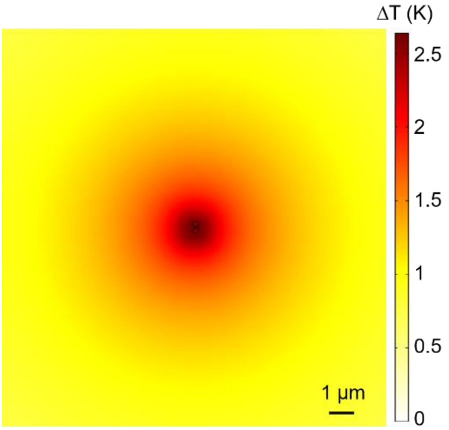

Supplementary Figure 4: thermal simulation of the same double nanohole geometry but sitting on top of glass substrate under the same illumination condition as mentioned in Fig. 4c, indicating the sapphire substrate we used in our experiment dissipate heat more efficiently.

Supplementary Discussion III. Fluorescence enhancement using nanohole array with circular lattice in gold film.

The fluorescence labelled nano-object is considered a dipolar emitter perpendicular to the gold film. Once a nanoparticle is captured by the GET trap, the dipole emission is redirected and funneled into a small divergence angle (< 10°), and beamed up into the direction perpendicular to the gold film. As depicted in Fig. 1e, we show the collimated far-field radiation.

To achieve this collimation, we design our nanohole array to have a circular lattice as shown in Fig. 1d. Compared with regular square lattice nanohole array, circular lattice nanohole array has better rotational symmetry, which allows better emission collimation[4]. Our circular lattice nanohole array supports surface plasmon polariton (SPP) mode, which is also considered the reason why the famous Bull's eye grating can enhance collection efficiency[4–6]. The in-plane propagating SPP mode subsequently scatters on periodic nanoholes, finally resulting in highly directional emission, as shown in Supplementary Fig. 5.

Supplementary Fig. 6 shows the results of fluorescence emission enhancement using our optimized circular lattice nanohole array versus other non-optimized structures. The optimized nanohole array collimates the emission into a narrow radiation angle, as shown in the middle panel of Supplementary Fig. 6a, while the non-optimized ones support wider divergence angle. We investigate the average fluorescence intensities of trapped particles for different designs. It shows that the particles trapped by the optimized structure are brighter (Supplementary Fig. 6b and c). In the experiment, we use an objective lens of 40X magnification and NA = 0.75, which has a collecting angle indicated by the red dash lines in Supplementary Fig. 6a. The particle we used to initially quantify the emission enhancement factor is fluorescently-labeled mono-dispersed 100 nm polystyrene (PS) beads, which have emission spectrum matching the working range of our optimized circular lattice nanohole array. Using commercial labelled PS beads of identical size proves the ability of our circular lattice nanohole array to enhance fluorescence collection. Subsequently, the optimized design was used for trapping and enhancing the imaging of trapped EVs.

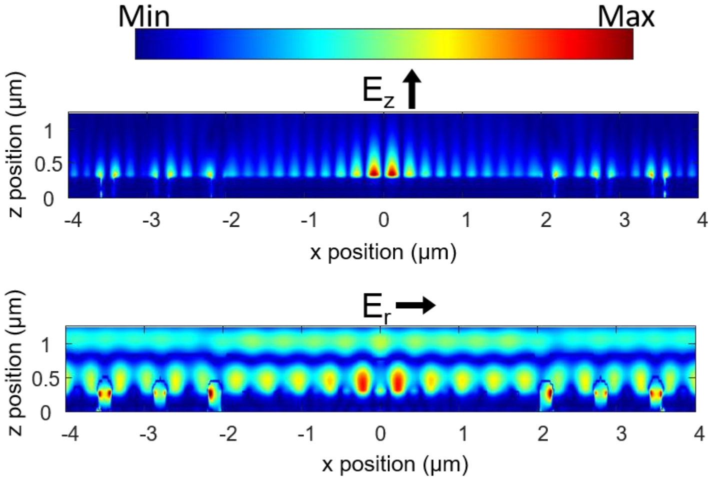

Supplementary Figure 5: out-of-plane (top) and radial (bottom) electric field distribution. It clearly shows the in-plane SPP mode and E_z components perpendicular to the surface.

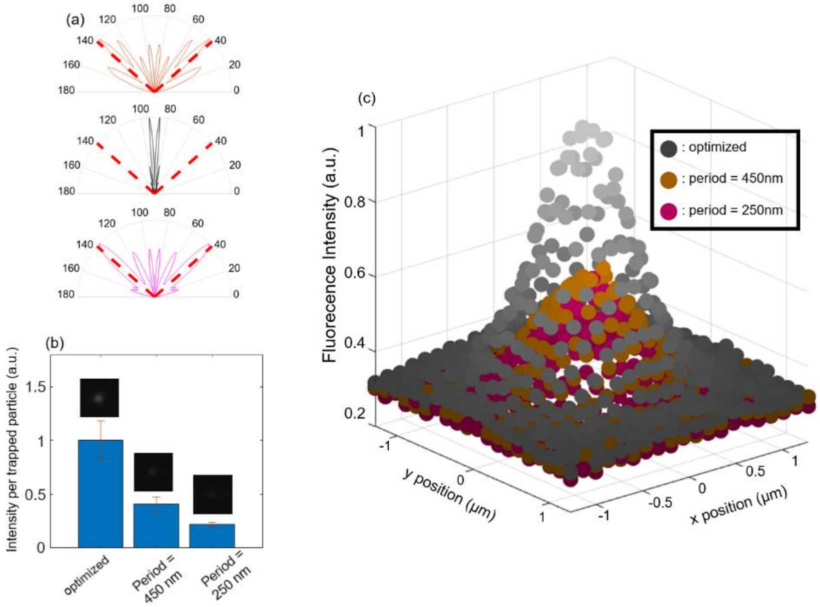

Supplementary Figure 6: (a) simulated far field radiation pattern simulated using FDTD method. Orange and magenta lines show the results of smaller and larger period, respectively. Black line shows the radiation pattern collimated by the optimized circular lattice design. Red dashed line indicates the angle corresponding to the NA of our objective lens. All numbers are in the unit of degree. (b) the experimental data showing the intensity per trapped particles normalized to the average intensity of on the optimized pattern. The insets show the typical fluorescence image of trapped particles on camera. (c) intensity maps corresponding to average intensity shown in (b). It clearly shows the fluorescence enhancement property of our optimized circular lattice nanohole array.

Supplementary Discussion IV. Filling factor under various particle concentration.

The percent ratio of particles trapping, as being called ‘filling factor’ is highly dependent on the concentration of particle. As shown in Supplementary Fig. 7, the larger the particle concentration is, the more GET traps are occupied as expected. It should be noted here that for particle concentration of 10^8 or

10^9 particles/mL, the number of available particles is larger than the number of GET traps in the field of view. Under this a.c. frequency of 2 kHz, more than one particle are captured within one GET trap.

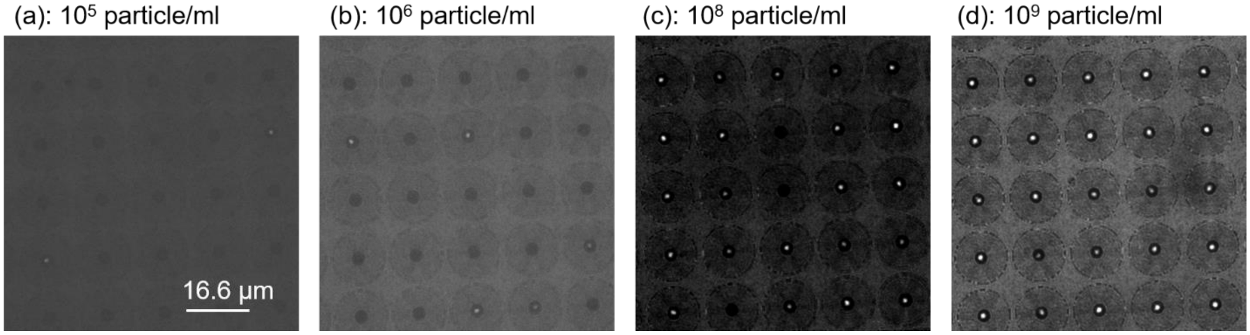

Supplementary Figure 7: (a) to (d), the fluorescence images showing the number of occupied GET traps under various 100 nm PS beads concentrations of 10^5,10^6,10^8 and 10^9 particles/mL, correspondingly. As the concentration of particle increases, more traps are occupied. The full video showing the parallel trapping under these four concentrations is provided in SIV 7.

Supplementary Discussion V. Dipole-dipole interaction force enables single particle resolution trapping.

We have performed detailed experiments to determine the conditions and optimal design of GET traps for achieving self-limiting single particle trapping in GET. To achieve self-limiting single particle resolution trapping, we harness the interplay between the in-plane drag force from a.c. electro-osmotic flow and the dipole-dipole repulsion force between particles. This self-limiting single particle-resolution trapping behavior persists for a wide range of particle concentration ranging from 10^4 to 10^8 particles/mL when the diameter of the void region is 4 μm. This frequency-dependent behavior is attributed to the fact that the dipole-dipole repulsion force between two particles overcomes the drag force from a.c. electro-osmotic flow. The a.c. electro-osmotic flow is frequency-dependent, and decreases for increasing a.c. field frequency, while the dipole-dipole repulsion force is weakly dependent on frequency as depicted in Fig. 2c. We attribute this weak dependence to the fact that when the a.c. frequency is low, the surface charge and double layer charge of the trapped particle have sufficient time to respond to and be polarized by the external a.c. electric field.

The diameter of the void region in GET is set to 4 μm. Supplementary Fig. 8 illustrates the forces on particles when more than one particle stays inside one trap.

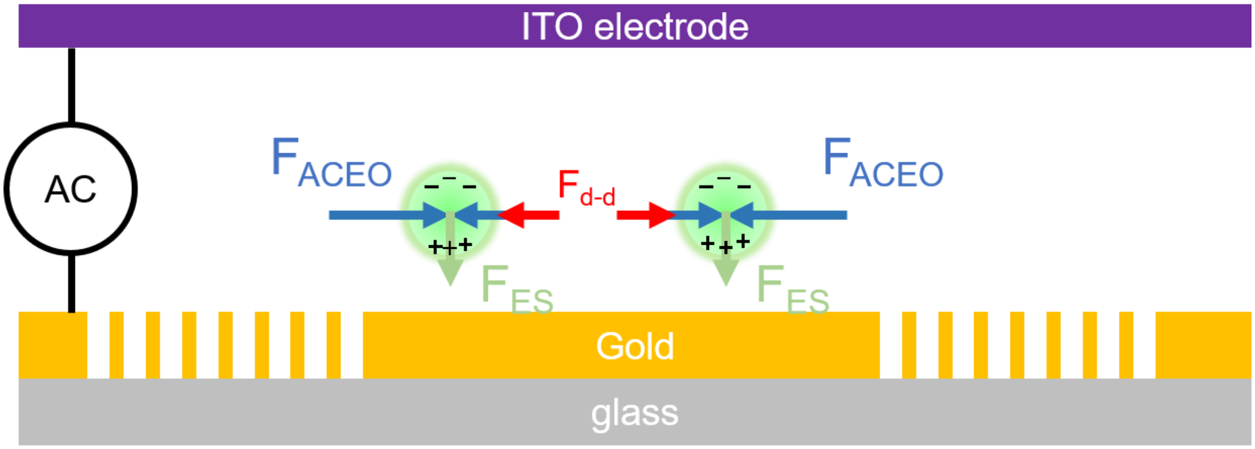

Supplementary Figure 8: force illustration shows the repulsive dipole-dipole interaction force when more than one particle stays inside one trap, in addition to the ACEO drag force and electrostatic force.

Due to the applied a.c. electric field perpendicular to the gold film, the surface charges on the particles are polarized along the vertical direction. Thus, the dipoles are pointing in the same direction, leading to a repulsive force in between particles[7,8]. The dipole-dipole interaction force can be explicitly express as[9]:

$$\mathbf{F}_{\mathbf{d-d}} = \frac{3}{4}\pi a^2 \varepsilon_m \alpha^2 \left(\vec{\mathbf{E}}_{\mathrm{rms}}\right)^2 \left(\frac{2a}{d}\right)^4 [(3\cos(\theta)^2 - 1)\hat{\mathbf{d}} + (\sin(2\theta))\hat{\boldsymbol{\theta}}]. \quad (8)$$

a is the particle radius, α is the particle polarizability, d is the center-to-center distance between particles and θ is the angle between particles with respect to the direction of electric field. $\vec{\mathbf{E}}_{\mathrm{rms}}$ is the root-mean-square amplitude of applied a.c. electric field. ε_m is the permittivity of medium at a.c. frequency.

It is also known that the effective polarizability of a nanometric particle is dependent on the electrostatic properties of the interface between the nanoparticles and the surrounding medium. In aqueous environment, the coating on colloidal nanoparticles can be interpreted as 'charge cloud', which is the so-called electric double layer (EDL)[10].

The zeta-potential indicates the properties of the EDL and the net (surface) charge of the colloidal particle, σ_s, through the equation given by[10,11]:

$$|\sigma_{surface}| = \frac{2\varepsilon_m \varepsilon_0 k_B T}{e\lambda_D} \left|\sinh\left(\frac{e\zeta}{2\phi_r}\right)\right|. \quad (9)$$

Here, e is the electron's charge, κ is the characteristic Debye length and ϕ_r is a minimum electrostatic energy that enable a stable formation of EDL, which is close to 25 meV at room temperature. The calculation of the Debye length is governed by:

$$\lambda_D = \sqrt{\frac{\mu \varepsilon_m \varepsilon_0 k_B T}{2 S_l z^2 e}}. \quad (10)$$

μ is the bulk mobility of water. z is the electrolyte charge number, which for DI-water medium is 1. S_l is the conductivity of water, which is measured using Zetasizer.

After obtaining the net surface charge, the next step is to estimate the conductivity inside the diffusion layer and it should have a higher conductivity than the bulk water. The total number of charges in the diffusion layer is estimated by:

$$N_d = N_{surface} = \frac{|\sigma_{surface}| \times 4\pi a^2}{e}. \quad (11)$$

Afterwards, the conductivity of the diffusion layer, the ‘charge cloud’ of colloidal particle, is obtained by:

$$S_c = \frac{N_d \Lambda}{4/3\pi((a+\lambda_D)^3 - a^3) \times N_A}. \quad (12)$$

Λ is the molar mobility of the ions inside diffusion layer, which is considered DI water here. N_A is Avogadro constant. To include the influence from the applied a.c. field and the high-conductivity coating layer, we write the complex permittivity in form of:

$$\varepsilon_{c/p}^* = \varepsilon_{c/p} + \frac{S_{c/p}}{j\omega}. \quad (13)$$

c/p stand for coating layer or particle, respectively. $\varepsilon_{c/p}$ is the permittivity of the coating layer or the particle and $S_{c/p}$ is the conductivity of either. $j = \sqrt{-1}$ and ω is the a.c. frequency.

Finally, we adapt the coating-layer-modified equation from reference[10] to represent the polarizability to finalize our calculation of the dipole-dipole interaction force:

$$\alpha_{NP} = 3(V_{NP} + V_c)\frac{(\varepsilon_c - \varepsilon_m)(\varepsilon_{NP} + 2\varepsilon_c) + f(\varepsilon_{NP} - \varepsilon_c)(\varepsilon_m + 2\varepsilon_c)}{(\varepsilon_c + 2\varepsilon_m)(\varepsilon_{NP} + 2\varepsilon_c) + f(2\varepsilon_c - 2\varepsilon_m)(\varepsilon_{NP} - \varepsilon_c)}. \quad (14)$$

Here, V_c and V_{NP} are the volume of coating layer and nanoparticle, respectively, and $f = V_{NP}/(V_{NP} + V_c)$. $\varepsilon_{c/NP/m}$ stands for the complex permittivity of coating/nanoparticle/medium. The dipole-dipole interaction force between two 100 nm PS beads of 500 nm distance away is presented in the main manuscript (Fig. 2c), which is quite constant within the operational range of a.c. frequency tuning utilized.

As discussed in the previous section, at trapping frequencies, the strength of the ACEO flow is greater as the a.c. frequency is lowered, while it reduces as the a.c. frequency increases. Hence, as the a.c. frequency is increased from 2 kHz to 3.5 kHz, repulsive dipole-dipole interaction force remains the same, but the drag force from the ACEO flow is weaker. The dipole-dipole interaction dominates over the drag force from ACEO flow to expel a second particle and allows only one particle to stay in the GET trap.

Supplementary Discussion VI. Trapping particles of various sizes on GET.

Since exosomes are heterogeneous nanosized biological particles with size ranging from 30 nm to 150 nm in diameter, in order to investigate the ability of GET to capture particles with various sizes, we conducted experiments on trapping PS beads with sizes of 200 nm and 20 nm in diameters. The results are shown below.

Initially, we test on PS beads with 200 nm in diameter, which is beyond the upper bound of commonly-known size of EVs. Supplementary Fig. 9 clearly shows the capability of GET to capture polystyrene beads.

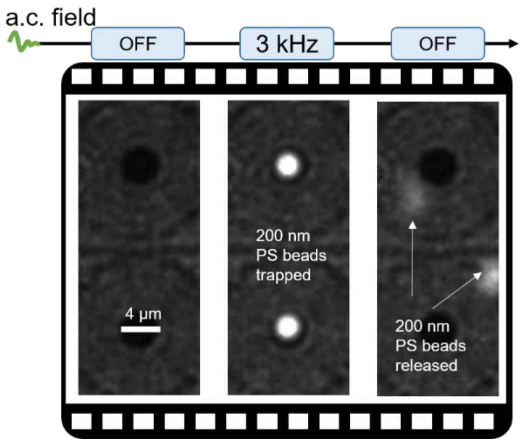

Supplementary Figure 9: 200 nm polystyrene beads are trapped by GET under 3 kHz a.c. frequency, as shown in SIV 8.

As illustrated in Supplementary Fig. 10, after 3 kHz a.c. electric field was turned on, 20 nm PS beads are trapped by the array of electrohydrodynamics potentials. The concentration of 20 nm PS beads is 10^8 particles/mL in the demonstration. Thus, most of the cavities are occupied by the PS beads.

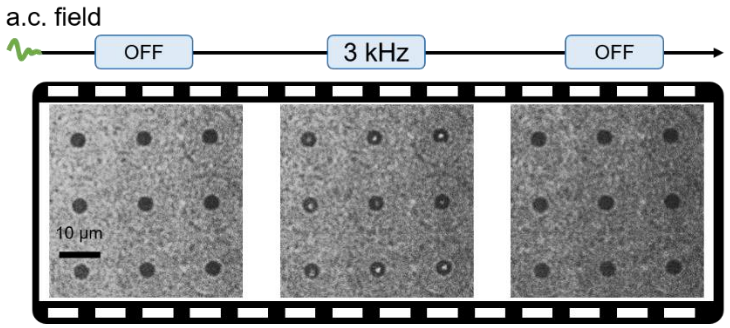

Supplementary Figure 10: 20 nm polystyrene beads are trapped by GET under 3 kHz a.c. frequency, as shown in SIV 9.

Supplementary Discussion VII. loading time versus particle concentration.

Here we conducted experiments for the comparison of loading time to a single DNH plasmonic cavity versus the loading time of GET traps. Over the particle concentration we tested (10^5 to 10^9 particles/mL), GET always enables rapid loading and finishes loading within 3 seconds. However, for a single DNH cavity (without GET), due to the lack of active approach to transport particles, the waiting time before trapping events happen is determined by the Brownian motion of particles. For the experiments, we used 100 nm polystyrene beads.

We can also theoretically estimate the waiting time under various particle concentrations for loading the trap based on free diffusion. To do so, the mean square diffusion distance is given by $R^2(t) = 2Dt$, and the diffusion coefficient $D = k_B T/6\pi\eta a$, where η is the viscosity of liquid and a is the radius of particle. Here, assuming room temperature (300 K) and viscosity of water $\eta = 8.9 \times 10^{-4}\ Pa \cdot s$. The radius of particle is 50 nm. The average diffusion distance is considered to be related to the concentration of particles.

Assuming the concentration is C_p and the distance between particles is large enough to avoid particle-particle interaction, we can calculate the average volume of solution occupied by each particle.

One molar solution contains 6.02×10^{23} polystyrene particles per liter, equal to 0.6 particles/nm^3. Thus, for the concentration of C_p, the average separation between particles is $R = 1.18/\sqrt[3]{C_p}$ [12]. We finally plot out the average diffusion time for a particle to visit the trapping site and get trapped.

Relying on Brownian motion, we conducted experiments to trap 100 nm PS of various concentrations, and the extracted time spans between turning on the laser to trapping event happening are plotted together with the estimation. Under 10^8 or 10^9 particle/mL concentration, trapping happened after 223 s and 36.9 s, respectively. But in a solution with a concentration of 10^5 particles/mL, we kept the laser on for one hour and trapping has not happened. In contrast, with the GET system, to trap 100 nm PS beads with concentration ranging from 10^5 to 10^9 particles/mL requires less than 5 seconds, as plotted in Supplementary Fig. 11. This comparison clearly proves the superiority of GET system to speed up the loading process.

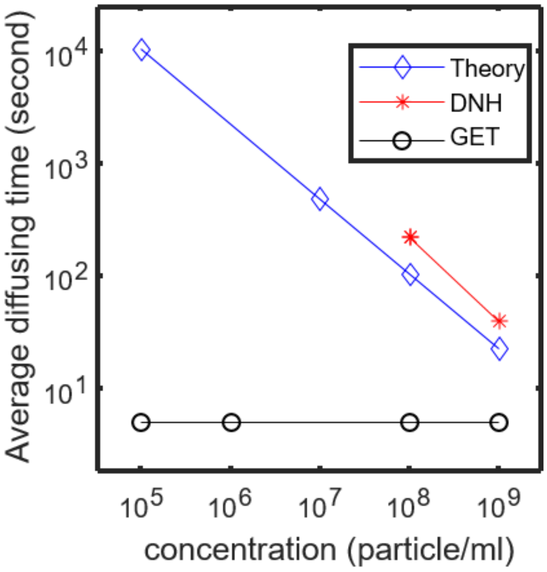

Supplementary Figure 11: the average diffusion time obtained from theoretical estimation (blue), single plasmonic cavity (red) and GET system (black). It clearly shows the GET can rapidly transport particles towards trapping site even at 0.16 femtomolar (10^5 particles/mL) concentration, which is not feasible for traditional optical tweezers or near-field tweezers.

Supplementary Discussion VIII. Dielectrophoresis

Dielectrophoresis (DEP) is the phenomenon where a dielectric particle experiences a force in a non-uniform electric field. Here, as the nanohole array perturbs the a.c. electric field to create ACEO flow, the distorted a.c. electric field may also exert DEP force onto trapped particles. DEP force is governed by:

$$\mathbf{F_{DEP}} = 2\pi a^3 \varepsilon_m Re\left(\frac{\varepsilon_p^* - \varepsilon_m^*}{\varepsilon_p^* + 2\varepsilon_m^*}\right)\nabla\left|\vec{\mathbf{E}}_{\mathrm{rms}}\right|^2, \quad (15)$$

where $\varepsilon_p^* = \varepsilon_p + \frac{S_p}{i\omega}$ and $\varepsilon_m^* = \varepsilon_m + \frac{S_m}{i\omega}$. ε_m and ε_p are the values of permittivity of the medium and particle, respectively. S_m and S_p are the conductivities of medium and particles, respectively. $\vec{\mathbf{E}}_{\mathrm{rms}}$ is the

root-mean-square value of the applied a.c. electric field and ω is the a.c. frequency. The term $\frac{\varepsilon_p^* - \varepsilon_m^*}{\varepsilon_p^* + 2\varepsilon_m^*}$ is also defined as the Claussius-Mossoti factor (CM factor), which determines if the DEP is repulsive or attractive. The value of the CM factor versus a.c. frequency is obtained from prior research[13], resulting in a shallow potential of 0.03 $k_b T$ from DEP. Compared with the ACEO potential from Stoke's drag, the influence from DEP can be ruled out from this analysis.

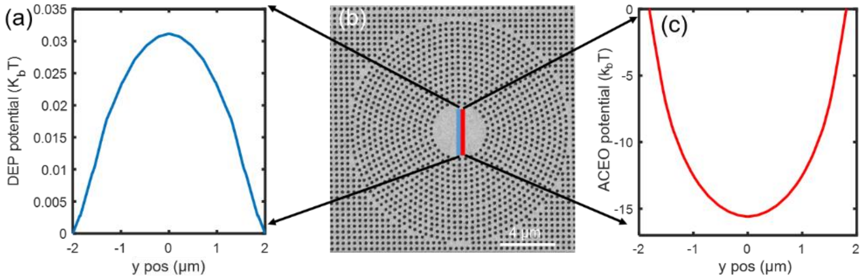

Supplementary Figure 12: (a) potential from DEP. (b) SEM of one GET trap. The blue line and red line show the positions where we extract the trapping potential in (a) and (c), respectively. (c) is the trapping potential from ACEO drag force.

Since the plasmonic cavity (DNH) may slightly perturb the a.c. electric field, we also studied the DEP force created in its presence. Using the same method described in SI section I for calculating the a.c. electric field, we map the in-plane DEP force exerted on a 100 nm polystyrene bead positioned 50 nm above the gold film using equation (15). The colormap in Supplementary Fig. 13 reveals that the DEP force is ~1.5 fN, which is negligible.

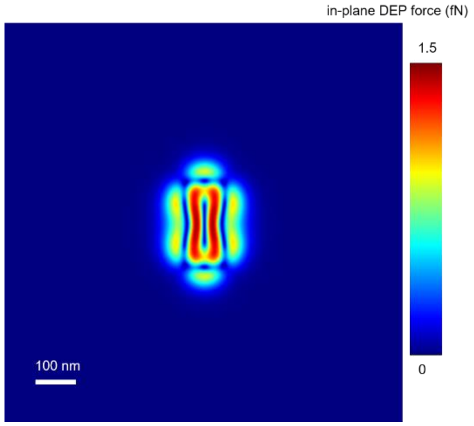

Supplementary Figure 13: the colormap showing the in-plane DEP force experienced by a 100 nm polystyrene bead situating 50 nm above the gold surface, calculated using equation 15.

Supplementary Discussion IX. Sample fabrication

In this study, there are two designs with gold film sitting on a glass or sapphire substrate. Sapphire substrate, which has a higher thermal conductivity relative to glass is used for plasmonic trapping in a GET system experiments to dissipate plasmonic heating.

For the plain GET trap, without superimposed double nanohole aperture, we employ the template strip method to transfer the nanohole array pattern into the gold film. Beginning with a bare 15mm by 15mm

silicon chip, Piranha solution and oxygen plasma were used to clean the surface of silicon chip. Next, a 10 nm Chromium hard mask was thermally evaporated onto the chip. We then spin-coated on PMMA A4 photoresist (PR) for e-beam lithography (EBL) to define the nanohole pattern. After development, a very short oxygen plasma (4 seconds) was applied to descum the developed PR. The sample was then soaked into Cr etchant (Transene) for 10 s, followed by a thorough DI water rinse. The nanohole pattern is thus transferred into Chromium, serving as reactive-ion etch (RIE) hard mask. The final RIE took ~ 1min to ensure that the depth of holes in silicon is greater than 500 nm and then the Cr hard mask was stripped off in Cr etchant to realize the silicon template.

The next step is to deposit a thin (120 nm) gold film directly onto the silicon template. A glass substrate coated with a UV curable epoxy was placed on the silicon template with gold film. After curing under UV light, the gold film was transferred to the glass substrate via template stripping process. The silicon template is reusable by cleaning in a gold etchant solution to remove the residual gold.

To properly dissipate excess heat, the gold nanohole array and double nanohole plasmonic aperture was also fabricated on a sapphire substrate with a high thermal conductivity of ~25 (W/m·K). This was achieved by deposition a thin film of gold on sapphire substrate and milling the gold nanohole array and double nanohole aperture plasmonic nanoantenna directly using focused ion beam milling.

References:

1. Hong, C., Yang, S. & Ndukaife, J. C. Stand-off trapping and manipulation of sub-10 nm objects and biomolecules using opto-thermo-electrohydrodynamic tweezers. *Nat Nanotechnol* **15**, 908–913 (2020).

2. Ndukaife, J. C. *et al.* Long-range and rapid transport of individual nano-objects by a hybrid electrothermoplasmonic nanotweezer. *Nat Nanotechnol* **11**, 53–59 (2016).

3. Ramos, A., Morgan, H., Green, N. G. & Castellanos, A. Ac electrokinetics: a review of forces in microelectrode structures. *J Phys D Appl Phys* **31**, 2338–2353 (1998).

4. Andersen, S. K. H. *et al.* Hybrid Plasmonic Bullseye Antennas for Efficient Photon Collection. *ACS Photonics* **5**, 692–698 (2018).

5. Choy, J. T. *et al.* Spontaneous emission and collection efficiency enhancement of single emitters in diamond via plasmonic cavities and gratings. *Appl Phys Lett* **103**, 161101 (2013).

6. Jun, Y. C., Huang, K. C. Y. & Brongersma, M. L. Plasmonic beaming and active control over fluorescent emission. *Nature Communications 2011 2:1* **2**, 1–6 (2011).

7. Work, A. H. & Williams, S. J. Characterization of 2D colloids assembled by optically-induced electrohydrodynamics. *Soft Matter* **11**, 4266–4272 (2015).

8. Mittal, M., Lele, P. P., Kaler, E. W. & Furst, E. M. Polarization and interactions of colloidal particles in ac electric fields. *J Chem Phys* **129**, 064513 (2008).

9. Ndukaife, J. C. *et al.* High-Resolution Large-Ensemble Nanoparticle Trapping with Multifunctional Thermoplasmonic Nanohole Metasurface. *ACS Nano* **12**, 5376–5384 (2018).

10. Rodríguez-Sevilla, P. *et al.* Optical Forces at the Nanoscale: Size and Electrostatic Effects. *Nano Lett* **18**, 602–609 (2018).

11. Kokot, G., Bespalova, M. I. & Krishnan, M. Measured electrical charge of SiO_2 in polar and nonpolar media. *J Chem Phys* **145**, 194701 (2016).

12. Erickson, H. P. Size and Shape of Protein Molecules at the Nanometer Level Determined by Sedimentation, Gel Filtration, and Electron Microscopy. *Biol Proced Online* **11**, 32–51 (2009).

13. Chen, Q. & Yuan, Y. J. A review of polystyrene bead manipulation by dielectrophoresis. *RSC Adv* **9**, 4963–4981 (2019).
