# Supplementary material for: Scalable trapping of single nanosized extracellular vesicles using plasmonics
Source: Nat Commun. 2023 Aug 9;14:4801. doi: 10.1038/s41467-023-40549-7 (PMC10412615; doi:10.1038/s41467-023-40549-7)
Supplement: Supplementary file 3 — Description of Additional Supplementary Files [file 41467_2023_40549_MOESM3_ESM.pdf]

Description of Additional Supplementary Files:

Supplementary Movie 1: Fast parallelized transport and trapping of nanoparticles using GET. Starting from vacant GET traps, we turned ON the a.c. field with the frequency set to 3.5 kHz and PS beads (100 nm) start to be rapidly transported and trapped across the multiple trapping sites on-chip in parallel within seconds. The AC field was kept ON for 2 minutes and the particles remained trapped. Then we turn OFF the a.c. field and immediately all the particles are released.

Supplementary Movie 2: Demonstrating self-limiting single-particle resolution trapping behavior. This video shows that at low a.c. frequency (2.5 kHz) and high particle concentration (100 nm PS beads with a concentration of 10^8 particles/mL), up to two particles were trapped in one of the GET trapping sites. To ensure only a single particle is trapped, we increased the a.c. frequency to 3.5 kHz to decrease the strength of the ACEO flow. The extra particle is released from the trap and only one single particle stays trapped under the 3.5 kHz a.c. frequency.

Supplementary Movie 3: Parallel trapping of single EVs. This video shows our GET trap can simultaneously capture individual exosomes of various sizes in parallel, with over 80% percent of the trapping sites filled with single exosomes within seconds.

Supplementary Movie 4: Instantaneous plasmon-assisted optical trapping in a GET system. At the beginning, the a.c. electric field is ON and several of single EVs are trapped by the electrohydrodynamic potential in parallel near each plasmonic cavities. Subsequently, a 6.3 mW laser is used to illuminate one of the double nanohole aperture antenna. This results in the superposition of the EHD trapping potential with a plasmon-enhanced optical trapping potential. Under the 6.3 mW laser power, the heating effect from laser is too weak to generate any ETP flow, while the optical force is strong enough to hold the EV with higher stability. As the a.c. field is turned off, all other EVs are released but the one under laser stays trapped by the plasmon-enhanced optical trapping force. The a.c. field is turned ON again to hold the EVs with the EHD potential, while the laser focus is moved to another location.

Supplementary Movie 5: Dynamic manipulation with GET. We subsequently increased the laser power to 25 mW and focused on the gold nanohole array to ensure a decent strength of ETP flow due to the collective heating from the gold nanohole array. A nearby trapped EV is released from the trap by the induced ETP flow and relocated to another GET trapping site. Finally, the laser power is reduced to 6.3 mW and used to illuminate the double nanhole aperture antenna to trap the EV with plasmon-enhanced optical trapping force.

Supplementary Movie 6: Precise 'printing' of single EVs to plasmonic cavity hotspots. While holding an EV using plasmon-enhanced optical force in one of the double nanohole aperture antenna, a low frequency a.c. field of 100 Hz frequency is applied to immobilize the trapped EV precisely at the plasmonic nanogap hotspot.

Supplementary Movie 7: Trapping 100 nm polystyrene beads of various particle concentration (10^5 to 10^9 particles/mL) with GET. Starting with vacant GET traps, as the a.c. electric field was turned on, at 2 kHz, 100 nm PS beads were rapidly loaded into the array of electrohydrodynamic traps of GET. The loading speed is independent to the concentration of particles (because every particle visits the nearby GET site in the array), but the 'filling factor' is larger as the concentration of particle increases. At 10^5 particles/mL solution, only 2 traps were occupied; but at 10^9 particles/mL concentration, all the traps were occupied with beads.

Supplementary Movie 8: Trapping 200 nm polystyrene beads with GET. At the concentration of 10^7 particles/mL, GET successfully trapped 200 nm polystyrene beads.

Supplementary Movie 9: Trapping 20 nm polystyrene beads with GET. At the concentration of 10^8 particles/mL, GET successfully trapped 20 nm polystyrene beads within seconds.

Supplementary Movie 10: No trapping observed on a single plasmonic cavity without ACEO flow under 10^5 particle/ml particle concentration. With particle concentration of 10^5 particles/mL, we kept the laser on for 1 hour but no trapping was observed.

Supplementary Movie 11: Loading a single plasmonic cavity without ACEO flow under 10^8 particle/ml concentration. In solutions with 10^8 and 10^9 particles/mL, we observed the trapping event happens in 223 s, after the laser was turned on.

Supplementary Movie 12: Loading a single plasmonic cavity without ACEO flow under 10^9 particle/ml concentration. In solutions with 10^8 and 10^9 particles/mL, we observed the trapping event happens 39.6 s, after the laser was turned on.

Supplementary Movie 13: Long-lasting trapping without damage using GET. This video demonstrates the rapid load to a plasmonic cavity and near-field trapping on the plasmonic cavity. After the particle was captured, we kept the laser on and observed the particle after 25 min. The fluorescence emission did not change after 25 min trapping, indicating no structural damage on the particle.
